# Acute dilated cardiomyopathy in the setting of catastrophic antiphospholipid syndrome and thrombotic microangiopathy: A case series and review

**DOI:** 10.1002/jha2.71

**Published:** 2020-07-31

**Authors:** Melody Hermel, David Hermel, Saif Azam, Jerold Shinbane, Annahita Sarcon, Erika Jones, Arjun Mehta, Luanda Grazette, Howard Liebman, Ilene Weitz

**Affiliations:** ^1^ Department of Medicine Keck School of Medicine University of Southern California Los Angeles California; ^2^ Department of Cardiology Keck School of Medicine University of Southern California Los Angeles California; ^3^ Department of Pathology Keck School of Medicine University of Southern California Los Angeles California; ^4^ Jane Anne Nohl Division of Hematology Keck School of Medicine University of Southern California Los Angeles California

**Keywords:** atypical hemolytic uremic syndrome, cardiomyopathy, catastrophic antiphospholipid antibody syndrome, complement, thrombotic microangiopathy

## Abstract

Catastrophic antiphospholipid antibody syndrome (CAPS) is a rare form of antiphospholipid syndrome, an autoimmune condition characterized by vascular thromboses, pregnancy loss, and antiphospholipid (aPL) antibodies. Diagnosis of CAPS relies on thrombosis of at least three different organs systems over 1 week, histopathological evidence of small vessel occlusion, and high aPL antibody titers. In a subset of precipitating circumstances, activation or disruption of endothelial cells in the microvasculature may occur along with cardiomyopathy. We present two cases of CAPS‐associated dilated cardiomyopathy at our institution, focusing on disease management, pathophysiology, and treatment. These patients were of Southeastern Asian descent, raising the possibility of genetic polymorphisms contributing to the development of cardiomyopathy. Both met CAPS criteria and both demonstrated clinicopathologic thrombotic microangiopathy (TMA) and complement activation and developed severe dilated cardiomyopathy with shock. Complement activation plays an important role in the development of CAPS and may be important in the pathogenesis of CAPS‐associated cardiomyopathy. Clinical suspicion for TMA as a pathophysiologic mechanism of unexplained heart failure in CAPS is important and increased awareness of cardiac side effects is necessary so that early treatment can be initiated to halt further cardiac and systemic complications.

## 1 INTRODUCTION

Antiphospholipid antibody syndrome (APLS) is a systemic, autoantibody‐mediated disease characterized by venous and/or arterial thrombosis, obstetric complications [1], and persistently detectable serum titers of antiphospholipid (aPL) antibodies (specifically anti‐β_2_ glycoprotein 1, anticardiolipin, and/or lupus anticoagulant antibodies) [2, 3, 4]. In less than 1% of patients with APLS, a life‐threatening complication known as catastrophic aPL antibody syndrome (CAPS) can develop in the presence of precipitating circumstances such as infection [5, 6], neoplasm [7], anticoagulation withdrawal [8], and pregnancy [9, 10]. Although diagnosis can be clinically challenging, international consensus criteria exist specifying diagnosis based on thrombosis of at least three different organs systems over a period of 1 week, confirmed aPL antibodies, and histopathological evidence of multiple small vessel occlusions [11, 12]. CAPS is thought to be due to the pathogenic effects of aPL on the vascular endothelium [13]. This can lead to thrombotic microangiopathy (TMA) characterized by development of fragmentation hemolytic anemia, thrombocytopenia, and organ dysfunction due to small vessel thrombosis, resulting in ischemia [14].

As a multisystem thrombophilic disorder, CAPS often manifests clinically with significant cardiac disease. In a study of 500 patients from the International CAPS Registry, 50% of patients with CAPS had a component of cardiac disease involvement [15]. In the majority of these cases, the pathologic findings showed evidence of myocardial infarction and valvular disease. In addition, Libman‐Sacks endocarditis has been reported in 13% of CAPS patients with heart involvement and may be accompanied by stroke [16, 17]. Cardiomyopathy is less frequently described, but may develop as a consequence of coronary artery occlusion or significant valvulopathy [18]. However, rare case reports have identified CAPS patients with dilated cardiomyopathy, normal coronary angiogram, negative viral serologic tests, and no other identifiable causes of heart failure on routine testing [19, 20, 21, 22, 23]. In this cohort of patients, endomyocardial biopsy can demonstrate clinicopathologic features of TMA. This subset is especially noteworthy because of the difficulty in diagnosis, unique pathophysiologic mechanisms at play, and potential reversibility of underlying myocardial injury with therapy directed at suppressing overactivation of the complement system [24, 25, 26]. We present two cases of CAPS at our institution in whom diffuse cardiomyopathy with evidence of TMA was identified. The relevant literature is reviewed, and the role of complement inhibition in the treatment of certain subtypes of cardiomyopathy due to TMA is discussed.

## 2 PATIENT 1

**FIGURE 1:**
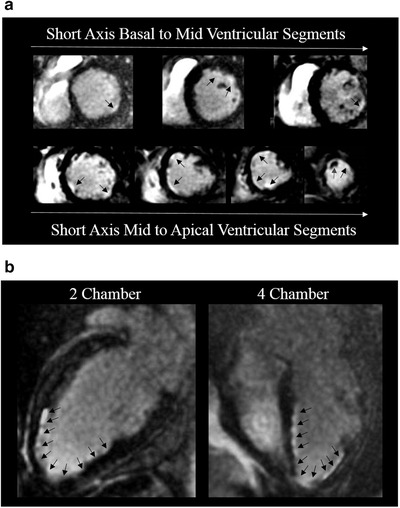
Patient 1 cardiac MRI. A, Serial short axis delayed contrast enhancement images from base to apex. Black arrows demonstrate areas of late gadolinium enhancement. Purple arrow demonstrates an apical thrombus. B, Left panel: Four chamber delayed contrast enhancement image; right panel: Two chamber delayed contrast enhancement image. Black arrows demonstrate areas of late gadolinium enhancement

**FIGURE 2:**
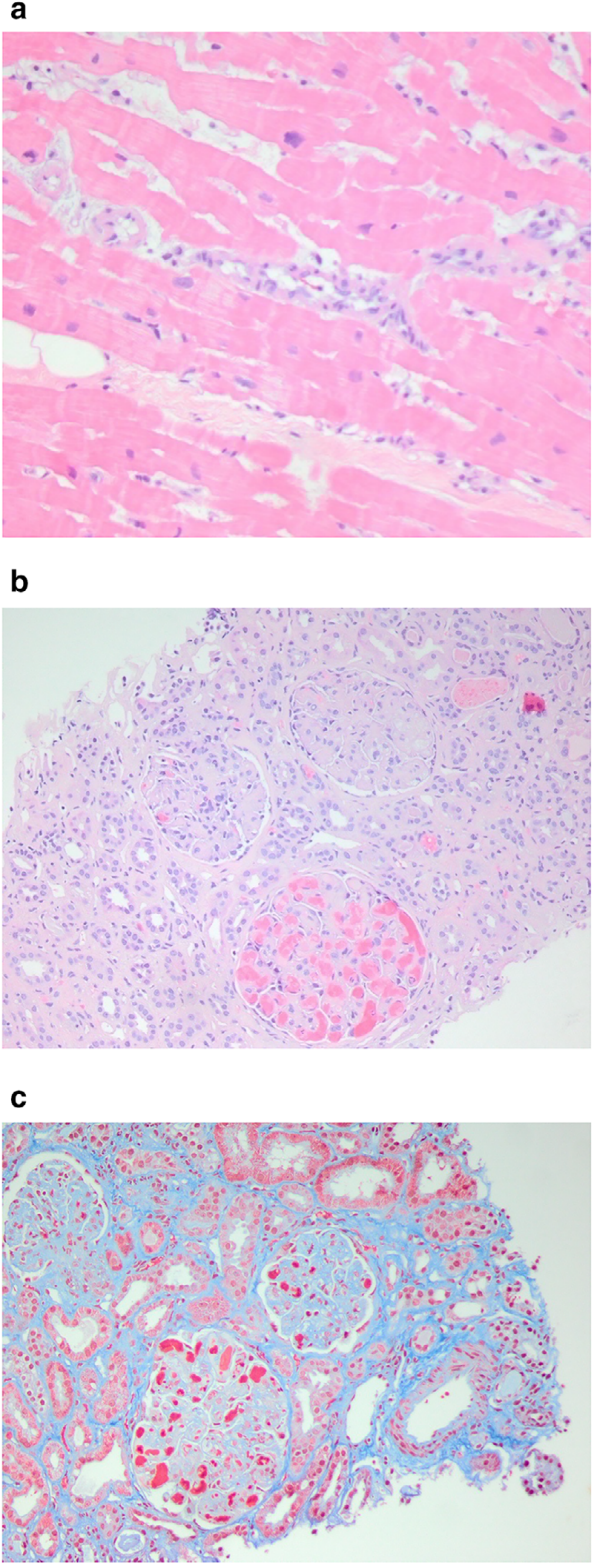
Patient 1 Pathology findings. A, HE heart myocyte: H&E‐stained section displays myocyte disarray and hypertrophy. B, HE kidney: H&E‐stained section demonstrates marked mesangial expansion, glomerular congestion, and fibrin thrombi within the glomeruli and arterioles. C, Trichrome kidney: Trichrome stained sections display glomerular congestion, mesangial expansion, fibrin thrombi, interstitial fibrosis and tubular atrophy, and arteriolar medial hyperplasia

A 34‐year‐old Malaysian woman presented for abdominal pain after a recent cholecystectomy for necrotizing cholecystitis. Incidentally, a small circumferential pericardial effusion was identified, prompting a transthoracic echocardiogram that revealed severe global hypokinesis, inferior wall hypokinesis, and a significantly reduced ejection fraction (25‐30% from a baseline of 40%). Her electrocardiogram (EKG) showed nonspecific T wave abnormalities. After a week of antibiotics for presumed cholangitis, she developed chest pain. The ensuing work‐up was notable for a computerized tomography (CT) pulmonary angiogram demonstrating new diffuse low density/hypoenhancement of the left ventricular subendocardium, thought to be secondary to myocarditis or multivessel ischemia. Coronary angiogram showed normal coronary arteries. Cardiac magnetic resonance imaging (MRI) demonstrated extensive subendocardial late gadolinium enhancement of the left ventricle and a small thrombus in the left ventricular apex (Figures 1A and 1B) and patient was treated with unfractionated heparin drip. On serologic testing, the patient was found to have a positive lupus anticoagulant and an elevated anticardiolipin (47 SGU) and anti‐β_2_ glycoprotein (30 GPL) immunoglobulin G (IgG). Complement levels were normal (C3, 112 mg/dL; C4, 25.6 mg/dL), anti‐Smith/U1‐RNP and anti‐double stranded DNA were negative, and antinuclear antibody testing was positive at a titer of 1:640. Despite antibiotics, heart failure‐directed therapy, and immunosuppressive agents (including prednisone, mycophenolate, and eculizumab), the patient returned weeks later with worsening dyspnea at rest, new proteinuria, oliguria, and an increased creatinine. Right ventricular endomyocardial biopsy was performed and showed nonspecific myocyte disarray and hypertrophy, though it was not diagnostic (Figure 2A). Given worsening renal function requiring initiation of hemodialysis, a renal biopsy was performed and found to be consistent with TMA (Figures 2B and 2C). von Willebrand factor cleaving protease (ADAMTS13) metalloprotease activity was normal at 151%. The patient was subsequently started on eculizumab 900 mg IV weekly with meningococcal prophylaxis. No complement regulatory gene mutations were identified, and later eculizumab efficacy monitoring with total hemolytic complement testing (CH_50_) indicated appropriate complement blockade.

Two months later, the patient returned with severe sepsis and consolidative lung opacities. She also had an acute change in mental status with MRI of the brain showing bilateral acute infarcts in the anterior and posterior circulations at gray‐white junctions. Lumbar puncture was negative for infection and cardiovascular workup was negative for a thrombotic nidus. The patient was subsequently found to be in status epilepticus and started on antiseizure medications, with a complicated hospital course marked by numerous pulseless electrical activity (PEA) arrests and intubation episodes. The patient had an enlarging area of discoloration to her left wrist with a tissue biopsy demonstrating microvascular thrombosis suggestive of TMA. She received IVIG and rituximab in addition to her continued eculizumab therapy with temporary improvement in her cardiomyopathy allowing for discontinuation of inotropes. Her subsequent hospital course was complicated by pneumonia, acute pancreatitis, and hypotension during hemodialysis requiring vasopressor support. Clinical findings included an elevated brain natriuretic peptide (BNP) greater than 5000 pg/mL, elevated troponin‐T up to 0.178 ng/mL, and right heart catheterization hemodynamic values consistent with cardiogenic shock. The last reported echocardiogram showed a markedly decreased ejection fraction of less than 20%. Ultimately, given the patient's condition, her family chose to transition to palliative care, and she expired.

## 3 PATIENT 2

A 57‐year‐old Thai man with a prior history of SLE and APLS (positive lupus anticoagulant, anticardiolipin [IgA 21 APL and IgG 105 GPL], and anti‐β_2_ glycoprotein [IgM 21 SAU and IgG 104 SGU]) complicated by stroke, submassive pulmonary embolism, liver infarct, Libman‐Sacks endocarditis (on therapeutic enoxaparin), ITP (status post IVIG and four cycles of cyclophosphamide, rituximab, dexamethasone, prednisone, and romiplistin), lupus cerebritis (complicated by psychosis and seizures), and chronic kidney disease presented with right upper quadrant abdominal pain and right flank pain for 6 days as well as acute encephalopathy. He became acutely altered, and a head CT was negative for hemorrhage or large infarct. Due to abdominal pain with worsening abdominal exam and history of APLS, there was concern for splanchnic vessel thrombosis and ischemia. Serology demonstrated an ANA titer of 1:80 (speckled pattern), negative dsDNA, negative ANCA, normal complement levels (C3, 104 mg/dL; C4, 25.9 mg/dL), and ADAMSTS13 metalloprotease activity abnormal at 34%. CT angiography of the chest, abdomen, and pelvis showed new bilateral pulmonary emboli, liver infarcts, and splenic infarcts. There was no evidence of renal involvement based on duplex ultrasound or CT angiography. However, high suspicion of CAPS‐associated TMA remained given the clinical finding of acute anuric renal failure. The patient was started on continuous renal replacement therapy. Given concern for CAPS versus ITP, therapy was started with IVIG × 5 days, pulse steroids, an unfractionated heparin drip, and eculizumab. Approximately 10 days after hospitalization, the patient experienced a rapidly dropping hemoglobin and hypotension. CT of the abdomen revealed retroperitoneal hematomas for which he underwent gel foam embolization of the right lumbar arteries with stasis of bleeding. Kidney function improved and the patient responded well to diuresis. The hospital course was further complicated by gram‐negative rod (*Bacteroides thetaiotaomicron*) bacteremia, *Clostridium difficile* infection, and *Pseudomonas aeruginosa* urinary tract infection treated with antibiotic therapy. The patient's anticoagulation was transitioned from heparin to dalteparin. He returned home after his prolonged hospital course with a plan for bimonthly eculizumab. Of note, this patient was found to have complement mutation Factor I (homozygous) missense c.1217G>A, p.Arg406His; CFHCHr1:196620917 C>T exon 18 c2808G>T, p.GLN672GLN exon 13 2016A>Gp.GLN672GLN. He did not require dialysis and showed clinical improvement allowing hospital discharge.

Several months after hospital discharge, the patient was noted to have worsening orthopnea. Transthoracic echo performed at that time revealed a severely reduced ejection fraction (26% from baseline of 50‐55%) with moderate diffuse hypokinesis with severe hypokinesis of the mid‐inferolateral and apical lateral myocardium, grade II diastolic dysfunction, mild to moderate increased size of the right ventricle with reduced systolic function, apical tenting of the mitral valve consistent with cardiomyopathy, and moderate to severe mitral and tricuspid regurgitation. Right heart catheterization showed elevated right and left heart filling pressures and a reduced cardiac index. Coronary angiogram showed evidence of single vessel coronary artery disease of the first diagonal branch, but the patient's heart failure was noted to be out‐of‐proportion to the mild degree of coronary artery disease. Additional findings included elevated troponin‐T of 0.16 ng/mL and an elevated pro‐BNP at 23 306 pg/mL in the absence of significant EKG changes. Cardiac MRI revealed mild left ventricular enlargement with severe global hypokinesis (EF 26%), normal right ventricular size with mild global hypokinesis, severe mitral regurgitation, severe aortic regurgitation, mild tricuspid regurgitation, mild aortic root ectasia, and no late gadolinium enhancement. He was discharged with guideline‐directed heart failure therapy and continued on his eculizumab, steroids, and dalteparin.

## 4 DISCUSSION

CAPS is a severe and rare complication of APLS with multiorgan thrombosis frequently involving the kidneys, lungs, brain, and heart [27, 28]. Here, we described two patients treated at our institution with CAPS and clinicopathologic signs of TMA who developed severe dilated cardiomyopathy and cardiogenic shock. Mechanisms of TMA, including complement activation, may have played an important role in the pathogenesis of their CAPS‐associated cardiomyopathy.

Both of our patients met CAPS criteria with three or more organ systems involved, progression of disease within 1 week, positive aPL antibodies, and clinicopathologic evidence of TMA in those biopsied. For each of our patients, other confounding TMA syndromes, including *Shiga* toxin‐induced hemolytic uremic syndrome (HUS) and thrombotic thrombocytopenic purpura (TTP), were ruled out on the basis of negative *Shiga* toxin and normal ADAMTS13 activity, respectively.

Of note, both of our patients were of Southeastern Asian descent, raising the possibility of additional genetic polymorphisms contributing to the development of cardiomyopathy. Both developed acute severe diffuse global hypokinesis as noted on an echocardiogram. Coronary angiogram was unrevealing as a source for the cardiomyopathy in both cases. On cardiac MRI, patient 1 had extensive left ventricular subendocardial late gadolinium enhancement with a small thrombus in the apex. Both patients had central nervous system involvement. Patient 2 had evidence of a mutation in the complement pathway, whereas the other patient did not undergo genetic testing. CH_50_ and genetics were done by outside labs. Mutation analysis for complement‐regulatory protein abnormalities was done by Machaon Diagnostics. Genomic DNA was extracted from patient whole blood samples. The genomic DNA was used as a template for a highly multiplexed polymerase chain reaction scheme designed to specifically amplify the exons, splice sites, and untranslated regions, in addition to several deep intronic and promoter sites, of the 12 genes of interest (CFH, CFI, CFB, CFHR1, CFHR3, CFHR4, CFHR5, CD46/MCP, C3, THBD, PLG, and DGKE). These polymerase chain reaction amplicons were further processed into libraries for sequencing on a next‐generation sequencer. Sequence data were aligned to the human reference genome (Hg19) to identify nucleotide variants. The variants were checked against private and public databases (1000 Genomes and www.fh-hus.org, e.g.) to help with interpretation. Patients were also screened for inhibitory antibodies against complement factor H (CFH). Both patients were treated with eculizumab, and improvement was seen in clinical and laboratory parameters. Based on hemodynamic findings, both patients suffered from a component of cardiogenic shock requiring vasopressors and inotrope therapy.

The incidence of cardiomyopathy in association with TMA is not well studied. In a retrospective study of 220 adult patients diagnosed with TMA at the Mayo Clinic, the estimated incidence of heart failure, defined by strict Framingham criteria, was roughly 10% and was associated with increased mortality [29]. The etiology of heart failure, however, was not specified. In a study of 17 autopsied patients with TTP, 13 had evidence of extensive small‐vessel thromboses within the heart arterioles [30]. Case reports also describe cardiomyopathy associated with TTP in CAPS, with two of three specifically showing improvement with treatment of the underlying disease, including plasmapheresis [31, 32, 33]. Likewise, in *Shiga* toxin‐producing *Escherichia coli*‐associated HUS, there have been described cases of dilated cardiomyopathy occurring in children and adults, with favorable cardiac outcomes after treatment [34, 35]. Furthermore, patients with cardiomyopathy secondary to complement‐mediated TMA, including those with atypical HUS and those with known complement Factor H gene mutations or autoantibodies directed against Factor H, showed clinical response to eculizumab treatment with improvement in their cardiomyopathy [36]. Cases of cardiomyopathy and complement‐mediated TMA are additionally described in the setting of preeclampsia‐induced premature delivery [9, 10, 37] and in diverse settings in the pediatric population [38, 39] with improvement with eculizumab [40]. Interestingly, Takotsubo cardiomyopathy has been described atypical HUS as well [41].

The role of CAPS‐related TMA and cardiomyopathy has not been frequently reported. In a single‐institution series of 14 patients with CAPS, one patient was reported to have cardiomyopathy associated with the syndrome, which was confirmed with endomyocardial histology showing small vessels with endoluminal thrombosis and perivascular/intramural inflammatory cells [42]. Although uncommon, there have been other reported cases of cardiomyopathy suspected due to CAPS‐induced TMA [43, 44, 45].

The mechanism of CAPS‐precipitated TMA and associated cardiomyopathy is thought to be an effect of aPL antibodies on endothelium integrity [46, 47, 48]. The large‐scale thrombosis occurs partly through a cytokine storm, propagated by an immune response to peptide sequences with structural homology to aPL antibodies, with subsequent increase in complement activation resulting in the release of pro‐inflammatory and prothrombotic cytokines that disrupt endothelial cell membranes and activate toll‐like receptors [6, 49, 55, 56, 57]. Thrombosis then leads to secondary consumption of protein C and antithrombin and an increase in plasminogen activator inhibitor type‐1 feeding a pro‐coagulant cycle. In addition, activation of platelets and increased expression of tissue factor further accelerates clot formation [13, 50]. Thrombin itself can enzymatically cleave C5 to C5a, inducing more cytokine activation as well as activation of more terminal complement through more C5b generation.

Recent studies also suggest evidence of complement activation in the thrombotic small vessels of patients with CAPS, possibly triggered by immune complex deposition of aPL antibodies. In vivo preclinical data suggest that in C3 or C5‐deficient mice, the effect of aPL antibody‐mediated thrombosis is attenuated in the same way as with a C5 inhibitor in wild‐type mice, suggesting that complement may be activated in patients with aPL antibodies resulting in excessive generation of C5a (which induces neutrophil and monocyte tissue factor‐dependent procoagulant activity as well as platelet aggregation and activation) [51]. This has ushered in a new treatment paradigm of TMA‐associated cardiomyopathy with C5 inhibitor eculizumab in addition to traditional use of anticoagulation, corticosteroids, plasma exchange, and IVIG [52, 53, 54].

Going forward, it is critically important to better understand the microvascular physiology and the role of complement in cardiomyopathy associated with CAPS TMA. Future registry participation and detailed mechanistic insight is needed to clarify this problem in rare diseases such as CAPS. One should have a high clinical suspicion for TMA as a pathophysiologic mechanism of unexplained heart failure in CAPS. Increased awareness about the potential cardiac effects in CAPS is necessary so that early treatment can be initiated in order to halt further cardiac and systemic complications.

## AUTHOR CONTRIBUTIONS

All authors helped with critical writing; conceptualized and designed the study; analyzed and interpreted the data; revised the intellectual content; and gave final approval for the version to be published.

## CONFLICT OF INTEREST

IW serves on a speakers’ bureau for Alexion Pharmaceuticals.
